# Supplementary material for: Projection-type see-through holographic three-dimensional display
Source: Nat Commun. 2016 Oct 3;7:12954. doi: 10.1038/ncomms12954 (PMC5063955; doi:10.1038/ncomms12954)
Supplement: Supplementary Information — Supplementary Figure 1 and Supplementary Notes 1 [file ncomms12954-s1.pdf]

Supplementary Figure 1

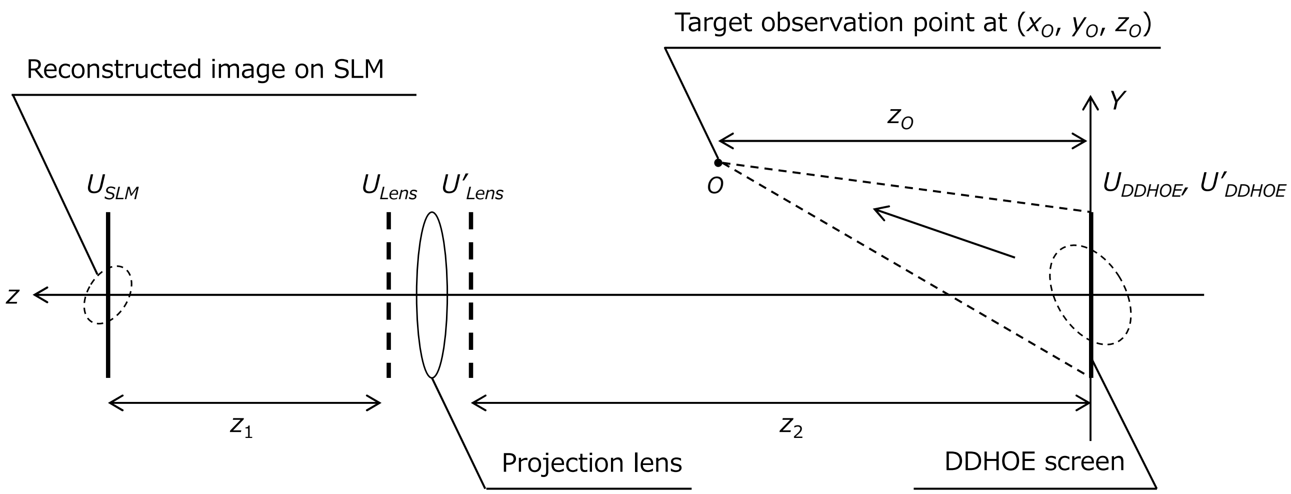

Supplementary Figure 1 | Simple model of the proposed system. The reconstructed wavefront on the SLM is largely projected on the digitally designed holographic optical element (DDHOE) screen by the projection lens, then reflected to the target observation point *O* via the phase modulation function implemented on the DDHOE screen.

Supplementary Note 1

In this note, the wavefront propagation from the holographic projector to the observable point via the digitally designed holographic optical element (DDHOE) screen is mathematically described along the optical path. A simple model of our display system is shown in Supplementary Figure 1. $U_{SLM}(u,v)$ is the field distribution of the reconstructed wavefront of the hologram data displayed on the spatial light modulator (SLM), which will be propagated to the projection lens. By using the Fresnel approximation formula, $U_{Lens}(x,y)$ is the incident wave distribution written as

$$U_{Lens}(x,y)=\frac{1}{j\lambda z_1}\iint_{-\infty}^{\infty}U_{SLM}(u,v)\exp\left\{j\frac{k}{2z_1}\left[(x-u)^2+(y-v)^2\right]\right\}dudv\,, \tag{1}$$

where k is the wave number and λ is the wavelength. Then, the projection lens with the focal length f_1 modulates the phase distribution of $U'_{Lens}(x,y)$ as

$$U'_{Lens}(x,y)=U_{Lens}(x,y)P(x,y)\exp\left[-j\frac{k}{2f_1}\left(x^2+y^2\right)\right], \tag{2}$$

$$P(x,y)=\begin{cases}1 & \textit{inside the projection lens aperture}\\ 0 & \textit{otherwise}\end{cases} \tag{3}$$

where f_1 is $z_1z_2/(z_1 + z_2)$ and $P(x,y)$ is the pupil function of the projection lens. After the propagation of $U'_{Lens}(x,y)$ to the DDHOE screen, the field distribution $U_{DDHOE}(x,y)$ on the DDHOE screen becomes

$$U_{DDHOE}(x,y)=\frac{1}{j\lambda z_2}\iint_{-\infty}^{\infty}U'_{Lens}(u,v)\exp\left\{j\frac{k}{2z_2}\left[(x-u)^2+(y-v)^2\right]\right\}dudv\,. \tag{4}$$

The DDHOE screen will then reflect the light with the addition of the phase function $\varphi(x,y)$ to concentrate the reflection light at the target observation point. The field distribution $U'_{DDHOE}(x,y)$ of the reflection light is written as

$$U'_{DDHOE}(x,y)=U_{DDHOE}(x,y)\exp\left[j\varphi(x,y)\right], \tag{5}$$

where the phase function $\varphi(x,y)$ in Eq. (5) is implemented on the DDHOE screen and can be written as

$$\varphi(x,y)=-\frac{k}{2f_2}\left(x^2+y^2\right)+\frac{k}{2z_P}\left(\xi^2+\eta^2-2\xi x-2\eta y\right), \tag{6}$$

where z_P is z_2-f_1 and f_2 is $z_Oz_P/(z_O + z_P)$, and ξ and η are

$$\xi=-\frac{z_P}{z_O}x_O,\quad \eta=-\frac{z_P}{z_O}y_O\,. \tag{7}$$

The reflection light $U'_{DDHOE}(x,y)$ on the DDHOE screen propagates distance z_O to the observation point as follows:

$$U_O(x,y)=\frac{1}{j\lambda z_O}\iint_{-\infty}^{\infty}U'_{DDHOE}(u,v)\exp\left\{j\frac{k}{2z_O}\left[(x-u)^2+(y-v)^2\right]\right\}dudv\,. \tag{8}$$

Finally, the observers at the target observable point $O = (x_O, y_O, z_O)$ will observe the reconstructed three-dimensional images over the DDHOE screen.
